# Comparison of photo‐matching algorithms commonly used for photographic capture–recapture studies

**DOI:** 10.1002/ece3.3140

**Published:** 2017-07-10

**Authors:** Maximilian Matthé, Marco Sannolo, Kristopher Winiarski, Annemarieke Spitzen ‐ van der Sluijs, Daniel Goedbloed, Sebastian Steinfartz, Ulrich Stachow

**Affiliations:** ^1^ Vodafone Chair Mobile Communication Systems Technical University Dresden Dresden Germany; ^2^ CIBIO, Research Centre in Biodiversity and Genetic Resources InBIO Universidade do Porto Campus de Vairão Vila do Conde Portugal; ^3^ Department of Environmental Conservation University of Massachusetts Amherst MA USA; ^4^ Reptile, Amphibian and Fish Conservation the Netherlands Nijmegen the Netherlands; ^5^ Department of Evolutionary Biology Zoological Institute Technische Universität Braunschweig Braunschweig Germany; ^6^ Leibniz Centre for Agricultural Landscape Research ZALF Müncheberg Germany

**Keywords:** AmphIdent, APHIS, capture–recapture, I3S, photographic identification, Wild‐ID

## Abstract

Photographic capture–recapture is a valuable tool for obtaining demographic information on wildlife populations due to its noninvasive nature and cost‐effectiveness. Recently, several computer‐aided photo‐matching algorithms have been developed to more efficiently match images of unique individuals in databases with thousands of images. However, the identification accuracy of these algorithms can severely bias estimates of vital rates and population size. Therefore, it is important to understand the performance and limitations of state‐of‐the‐art photo‐matching algorithms prior to implementation in capture–recapture studies involving possibly thousands of images. Here, we compared the performance of four photo‐matching algorithms; Wild‐ID, I3S Pattern+, APHIS, and AmphIdent using multiple amphibian databases of varying image quality. We measured the performance of each algorithm and evaluated the performance in relation to database size and the number of matching images in the database. We found that algorithm performance differed greatly by algorithm and image database, with recognition rates ranging from 100% to 22.6% when limiting the review to the 10 highest ranking images. We found that recognition rate degraded marginally with increased database size and could be improved considerably with a higher number of matching images in the database. In our study, the pixel‐based algorithm of AmphIdent exhibited superior recognition rates compared to the other approaches. We recommend carefully evaluating algorithm performance prior to using it to match a complete database. By choosing a suitable matching algorithm, databases of sizes that are unfeasible to match “by eye” can be easily translated to accurate individual capture histories necessary for robust demographic estimates.

## 1 INTRODUCTION

Understanding species population dynamics is an important step toward successful conservation. Capture–mark–recapture (CMR) and capture–recapture (CR) models have proven to be very useful for estimating population demography and for testing ecological hypotheses (Cormack, 1964; Jolly, 1965; Lebreton, Burnham, Clobert, & Anderson, 1992; Seber, 1965). CMR studies typically require invasive techniques (e.g., tags, toe‐clipping, visual implant elastomers, or insertion of passive integrated transponders) (Bailey, 2004; Guimarães et al., 2014; Winandy & Denoël, 2011). However, these invasive approaches can be cost prohibitive to implement and could potentially affect individual behavior or survival (Wilson & McMahon, 2004). Alternatively, many species have variable body markings that are individual‐specific (Arzoumanian, Holmberg, & Norman, 2005; Gamble, Ravela, & McGarigal, 2008; Karlsson et al., 2005) and can serve as a natural mark. Photographic CR exploits these natural markings and has evolved as a viable alternative to invasive techniques applied to a wide range of species (Arzoumanian et al., 2005; Bolger, Morrison, Vance, Lee, & Farid, 2012; Sacchi, Scali, Mangiacotti, Sannolo, & Zuffi, 2016).

Visually matching images of the same individual “by eye” is potentially feasible with hundreds of images, but is impractical with the large databases necessary to estimate vital rates or population size (Dunbar, Ito, Bahjri, Dehom, & Salinas, 2014; Gore, Frey, Ormond, Allan, & Gilkes, 2016; Kelly, 2001; Sacchi et al., 2016). Recently, photo‐matching algorithms have been developed and successfully applied to match images of unique individuals in large databases (Bolger et al., 2012). These methods are typically not fully automated and require the user to evaluate a number of top ranked matches (e.g., 10 or 20 images) based on a similarity score the algorithm calculates for all unique pairs of images (Crall, Stewart, Berger‐Wolf, Rubenstein, & Sundaresan, 2013; Morrison, Yoshizaki, Nichols, & Bolger, 2011). Several recent studies have evaluated the performance and effectiveness of available photo‐matching algorithms, but they are typically restricted to a single matching algorithm and image database [although see Morrison, Keinath, Estes‐Zumpf, Crall, and Stewart (2016)]. For a researcher to understand the limitations of different photo‐matching algorithms—and to be able to choose the best algorithm for the required purpose, a comparison of multiple photo‐matching algorithms and diverse databases is necessary. So, for all photographic CR studies over all taxa a proper evaluation of the appropriate algorithm is essential before the onset of the analysis.

Here, we compare the performance of four popular photo‐matching algorithms used in previous photographic CR studies of amphibians: Wild‐ID, I3SPattern+, APHIS, and AmphIdent. We consider amphibians to be a suitable object for case studies for the purpose of comparing photo‐matching algorithm performance as they often have large population sizes and many species exhibit individual external markings that make them suitable for individual recognition (Sacchi et al., 2016). However, we note that the used image matching algorithms are generally applicable to other taxa with similar spot patterns. Here, we compare the performance of the image matching algorithms using four amphibian databases of varying image quality (database size ranging from 2,197 to 12,488 images). We estimate recognition rates for each algorithm and evaluate the effects of database size and image characteristics. We focused our analysis on images with binary patterns (e.g., distinctive body markings that can be represented by only two colors) as these patterns are most common with herpetofauna and other wildlife which have unique individual markings (Drechsler, Helling, & Steinfartz, 2015; Speed, Meekan, & Bradshaw, 2007).

## 2 MATERIALS AND METHODS

### 2.1 Photo‐matching algorithms

In the present investigation, we compare two feature‐based and two pixel‐based photo‐matching algorithms. The feature‐based candidate algorithms Wild‐ID and I3S Pattern+ were chosen due to their popularity in the scientific community, although other feature‐based algorithms can be found in, for example, Crall et al. (2013); Lahiri, Tantipathananandh, Warungu, Rubenstein, and Berger‐Wolf (2011). AmphIdent and APHIS were chosen as the pixel‐based candidates as they have been applied to the largest databases among pixel‐based algorithms (Petrovska‐Delacretaz, Edwards, Chiassoli, Chollet, & Pilliod, 2014; Schoen, Boenke, & Green, 2015).

#### 2.1.1 Wild‐ID

The feature‐based algorithm of Wild‐ID (http://dartmouth.edu/faculty-directory/douglas-thomas-bolger) uses the scale‐invariant feature transform (SIFT) feature detector (Lowe, 2004) to find distinct features in a given image (Bolger et al., 2012). SIFT is useful for pattern matching as it is invariant to scale, viewpoint, rotation, and illumination, which cannot be completely mitigated with images of animals taken in the field. To evaluate the similarity of patterns in two images, the feature descriptors of both images are compared with regard to similar descriptors and geometrically consistent appearance. A similarity score is then calculated based on goodness of fit between the feature vectors of both images.

#### 2.1.2 I3SPattern+

Interactive Individual Identification System (I3S) (http://www.reijns.com/i3s) is a suite of different feature‐based pattern comparison algorithms specialized for certain types of patterns. I3S Pattern+ is optimized specifically to match binary patterns, where the binarization can be performed interactively. Similar to Wild‐ID, I3S Pattern+ relies on a feature descriptor [speeded‐up robust features (SURF) (Bay, Tuytelaars, & Van Gool, 2006)]. For each image pair, I3S Pattern+ determines key points in the pattern, based on the output of the SURF algorithm. I3S Pattern+ then calculates a similarity score based on how close key points of both images are to one another.

#### 2.1.3 AmphIdent

AmphIdent (http://www.amphident.com) uses a pixel‐based approach instead of a feature detector to calculate a similarity score for two images (Matthe, Schönbrodt, & Berger, 2008). Initially, each image is scaled down by 25% per dimension, assigning to the resulting pixels the average of the 4 × 4 original pixels. The similarity score for two images is based on the sum of the absolute differences of corresponding pixel values in both images. To improve robustness against translation, scaling and cropping differences, one image is scaled and translated by combinations of different scales and translations. The final similarity score is the maximum score calculated over all the investigated transformations. AmphIdent uses specialized modules for different amphibian species. However, all modules do apply the same general matching algorithm, and only differ in the way patterns are converted into binary images. Hence, only generally applicable matching algorithms are compared in this study.

#### 2.1.4 APHIS

APHIS (http://imedea.uib-csic.es/bc/ecopob/) implements two different matching algorithms (Oscar et al., 2015). One feature‐based approach which is similar to I3SPattern+; however, the key points are selected manually by the user. In this study, we focus on the second algorithm, where APHIS employs a pixel‐based approach, named image template matching (ITM) which uses the *matchTemplate* function of the Open Computer Vision Libraries (Itseez, 2016). This function slides one image over another to find the position where both images match best. Initially, the ITM method was proposed to match lizards *Podarcis muralis*, where the pattern area was equally split into three columns and two rows of patches, and the overall similarity score was the sum of the result of the *matchTemplate* function for each patch. Oscar et al. (2015) propose the ITM method for colored images, while pointing out that images with strong contrast perform better with the ITM method. Therefore, in this study, we investigate the ITM performance for both colored and binarized images. In principle, the technique of APHIS is similar to AmphIdent; however, in contrast to AmphIdent, it neither performs the 25% downscaling or scale optimization.

### 2.2 Image preprocessing

Several image preprocessing steps were performed on the databases prior to matching images with the photo‐matching algorithms. First, as all investigated algorithms rely on a consistent posture of the individuals, images of longish species (e.g., newts and salamanders) were straightened (Drechsler et al., 2015; Gamble et al., 2008). Specifically, this involved manually marking the spine of the individuals prior to an image operation which warps the spine to a straight line adjusting adjacent pixels to the spine accordingly. Second, a consistent rectangular region of the image was cropped to serve as the extracted pattern for the individual. Both actions did not require more than 30 s of manual operation.

Subsequently, for I3S, APHIS, and AmphIdent, images were binarized by a thresholding algorithm, that for I3S was manually aided and performed automatically for APHIS and AmphIdent, using the specific AmphIdent species module. For Wild‐ID, matching performance with colored patterns are reported, as matching with binary patterns resulted in inferior recognition rates.

Note that despite the amount of time spent on manually preprocessing large databases, a considerable time‐saving is achieved compared to manually matching all pairs of images. In particular, the time for computer‐aided matching grows linearly with the database size *N*, whereas as the number of pairs to compare manually is *N *× (*N *− 1)/2 (Arntzen, Goudie, Halley, & Jehle, 2004), the number of required manual comparisons grows quadratically (Table 1).

**Table 1:** Estimates of overall required processing time (hours) with manual and computer‐assisted matching, for different database sizes *N*. We assumed the manual preprocessing takes 30 s per image and a manual comparison takes 1 s. With computer aided matching, the top 10 ranking images are reviewed

*N*	100	500	1,000	5,000	10,000
Manual matching	1.4	34.6	138	347	1,339
Computer‐aided	1.1	5.6	11.1	55.5	111

### 2.3 Performance metrics

Algorithm performance was evaluated on images which were visually matched “by eye” in all of the databases. To estimate performance, only a representative subset of matching images in the database needs to be known. As false acceptance rate in photographic capture‐recapture is virtually zero (Petrovska‐Delacretaz et al., 2014; Sacchi et al., 2016), our analysis focuses on the recognition rate, that is, how well the algorithms manage to highly rank images that are known matches based on the similarity score. For each database, similarity scores were measured between all images with the three different photo‐matching algorithms. The rank of known matching images was then calculated based on all the other images in the database. For example, if the similarity score of a known match was higher than all other similarity scores in the database, the retrieved rank of the pair was 1. From the retrieved rank for all known matches in a specific database, their cumulative density function (CDF) *cdf*(*r*) was calculated. This CDF is a measure for the quality of the matches provided by the algorithm.

Specifically, the *cdf*(*r*) is defined as the number of known pairs that are ranked at *r* or better, divided by the overall number of known image pairs. For example, *cdf*(5) = 0.95 can be interpreted as meaning that 95% of all known matches are retrieved at rank 5 or better. The complementary CDF (CCDF) 1 − *cdf*(*r*) is the false rejection rate (FRR) when visually reviewing the *r* top ranked images.

To investigate the performance of each photo‐matching algorithm with different database sizes, we sampled smaller databases from the original databases by randomly selecting *x* images from the original databases and recalculated *cdf*(*r*) for the different database sizes. This procedure was repeated 50 times for each unique database size and the reported *cdf*(*r*) represents the average of those iterations.

The measure of the rank CDF describes the matching performance for a single matching image (e.g., a single recapture). With image databases that contain more than two images of the same individual, we also evaluated recognition rate with more than one matching image in the image database. We expected that performance would improve, as the photo‐matching algorithm has multiple chances of a matching image receiving a high similarity score.

### 2.4 Image databases

**Figure 1:**
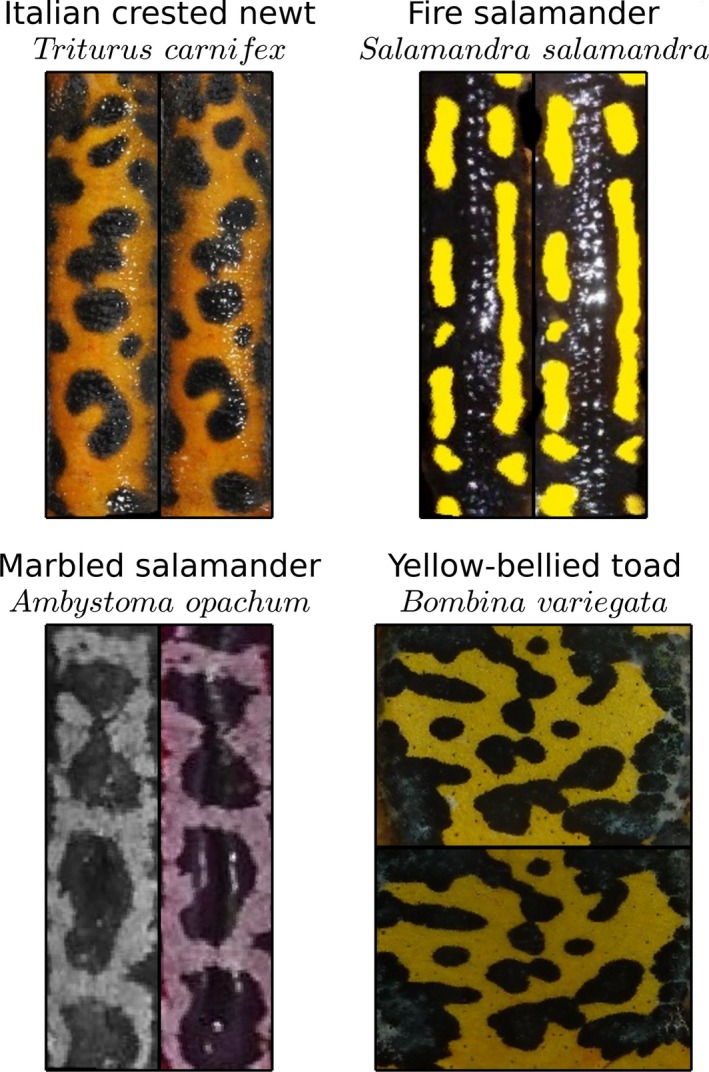
Representative images from the four investigated databases. For each species, two different images of the same individual are shown to highlight the slight differences in the pattern being matched

We analyzed four amphibian databases of varying image quality, that were used in previously published CR studies (Table 2, Figure 1). The databases were chosen to offer a large diversity of species, image qualities, and database sizes, limited by the accessibility of the images to the authors.

**Table 2:** Overview of image databases, preprocessing steps and image characteristics which differed by algorithm. Image dimensions are given in pixels and for APHIS, the number of patches for each pattern is provided in *italics font*

Species	Images	Straight?	Size Wild‐ID	Size I3S	Size AmphIdent	Size APHIS
Crested newt (Sannolo et al., 2016)	7,458	YES	320 × 1,280	320 × 1,280	80 × 320	300 × 1,200 *2 × 7 parts*
Fire salamander (Spitzen—van der Sluijs et al. unpublished data)	2,197	YES	320 × 1,280	320 × 1,280	80 × 320	300 × 1,200 *2 × 7 parts*
Marbled salamander (Gamble et al., 2008)	12,488	YES	320 × 1,280	320 × 1,280	80 × 320	300 × 1,200 *2 × 7 parts*
Yellow‐bellied toad (Neubeck & Braukmann, 2014; Schellenberg, 2016)	4,063	NO	960 × 800	960 × 800	240 × 200	480 × 400 *3 × 2 parts*

#### 2.4.1 Italian crested newt (*Triturus carnifex*)

**Figure 2:**
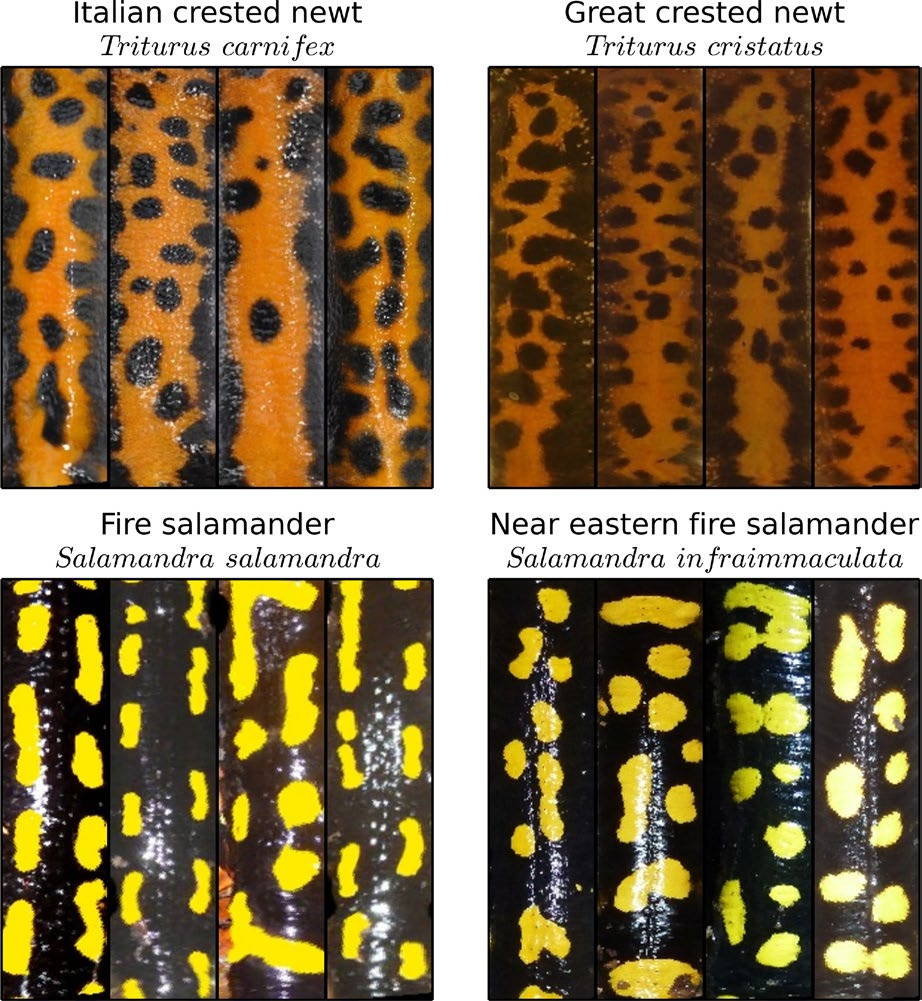
Representative patterns of *Triturus carnifex*,* Triturus cristatus*,* Salamandra salamandra,* and *Salamandra infraimmaculata*. Images of newts and salamanders have been merged into single databases. Compared to the Italian crested newt, patterns of the Great crested newt had smaller dots and finer structure

This database contained 672 images of the Italian crested newt (*Triturus carnifex*) Laurenti which were taken in 2014 from Groane Regional Park in northern Italy (Sannolo, Gatti, Mangiacotti, Scali, & Sacchi, 2016). Each time a newt was captured, it was photographed, then kept for one hour and photographed again to simulate a recapture. The database contained the simulated recaptures (386 matching image pairs); hence, all matches in this database were known. To increase overall database size, we merged this database with 6,787 images of the Great crested newt (*Triturus cristatus*) Laurenti, that were taken between 2006 and 2008 in an area 50 km east of Berlin, Germany (Berger, Graef, & Pfeffer, 2013; Matthe et al., 2008). The extracted patterns of *T. carnifex* and *T. cristatus* were similar, although *T. cristatus* had smaller spots and finer structure than *T. carnifex* (Figure 2). Before merging, we asserted that their subtle difference would not bias our performance analysis, by checking that the recognition rate for the known pairs was independent of the species in the database. Overall, these images were of high quality due to a consistent method of image acquisition (Matthe et al., 2008; Sannolo et al., 2016).

#### 2.4.2 Fire salamander (*Salamandra salamandra*)

This image database was obtained by merging two independent databases of the fire salamander (*Salamandra salamandra*) Linnaeus and the Near Eastern fire salamander (*Salamandra infraimmaculata*) Martens. The dataset included 446 images of individuals of a natural fire salamander population that were photographed between 2013 and 2015 in the Netherlands (Spitzen—van der Sluijs et al. unpublished data). The dataset was augmented by adding 1,751 images of *S. infraimmaculata*, which were taken between 2013 and 2014 in Tel Dan, Israel (Goedbloed et al., 2017). Similar to the newt dataset, it was visually confirmed that the patterns of both salamander species were similar enough to be merged into a single database. The salamander patterns consist of strips or roundish spots on the side of the back with a black area in the middle (Figure 2). The ground truth for this dataset was obtained by visual comparison of all 446 images (Spitzen—van der Sluijs et al., unpublished data). The analysis revealed 95 individuals that were captured between 2 and 16 times. Image quality was high in general; however, some images were impaired by significant glare in the center of the images.

#### 2.4.3 Marbled salamander (*Ambystoma opacum*)

This database contained 12,488 images of marbled salamanders (*Ambystoma opacum*) Gravenhorst, which were taken between 1999 and 2009 in western Massachusetts, USA. Detailed information about the capture study area and applied techniques for capturing are described in Gamble et al. (2008). Marbled salamanders exhibit patterns that are characterized by larger black areas in the center of the back which are separated by the brighter background. Ninety‐one known match pairs were found in the database by visual examination of a subset of the images. Manual comparison within a subset instead of the entire database was necessary, as the effort of comparison of all 12,488 images was unfeasible (cf. Table 1). Overall, image quality was fair due to low image resolution and occasional poor focus or glare.

#### 2.4.4 Yellow‐bellied toad (*Bombina variegata*)

This database contained 4,063 images of yellow‐bellied toads (*Bombina variegata*) Linnaeus and was merged from two independent databases. The yellow‐bellied toad patterns consist of smaller black spots that are distributed equally around the center area of the pattern. One of the merged databases consisted of 354 images and was collected in 2014 from Hainich National Park, Germany (Schellenberg, 2016). The other merged image database consisted of 3,709 images, collected from 2011 to 2013 in the area of Nordhessen, Germany (Neubeck & Braukmann, 2014). Special care was taken so that the images of both databases had the same quality and image properties. Overall image quality was high, although some images were degraded due to poor focus or glare. Known image matches were obtained by exhaustive visual examination of all images from Schellenberg (2016). The manual comparison revealed 83 distinct recaptured individuals, that were captured between two and ten times during the study.

## 3 RESULTS

### 3.1 Recognition rates by image database and photo‐matching algorithm

**Figure 3:**
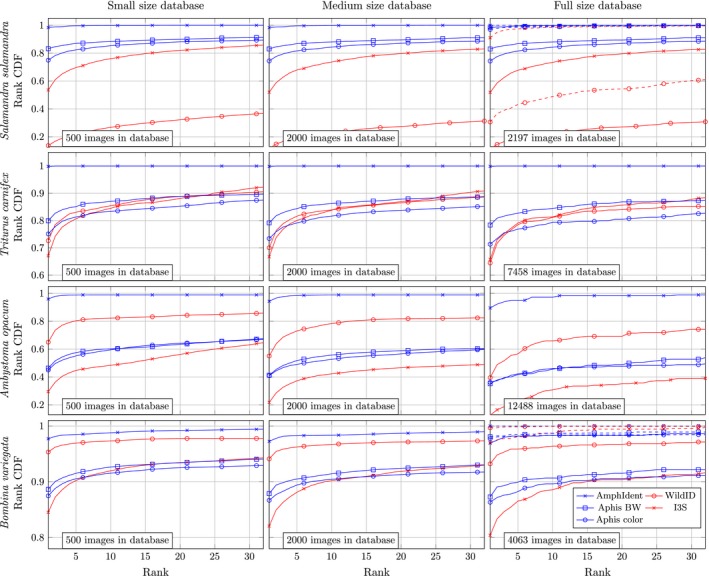
Rank CDFs for all algorithms and databases, by database size. Blue and red lines correspond to pixel‐based and feature‐based algorithms, respectively. Solid and dashed lines represent recognition rates with a single and three matching images in the database, respectively

We found that image‐matching performance differed between both algorithm and database, with AmphIdent performing best among all of the databases (Figure 3 and Table 3). The greatest performance differences between the algorithms by database was observed with the fire salamander database. Wild‐ID ranked 11.6% and 22.6% of all known matches as the top ranking image and among the top 10 ranked images, respectively. I3S ranked 51.9% and 73.4% as the top ranking image and among the top 10 ranked images, respectively. APHIS using color images ranked 74.4% and 84.3% of the known matches as the top ranking and the top 10 ranked images, and using binarized images 83.0% and 88.0% of the known matches were ranked top and within the top 10, respectively. AmphIdent ranked 98.3% and 99.8% of all known matches as the top ranking image and among the top 10 ranked images, respectively. The smallest difference in performance between the algorithms by species was observed with the yellow‐bellied toad database (Figure 3 and Table 3). Using Wild‐ID, 93.2% and 96.4% of the known matches were obtained at rank 1 and among the top 10, respectively. I3S ranked 80.4% and 88.6% of all known matches at the top and among the top 10 images, respectively. APHIS using color images ranked 86.3% and 89.6% at the top and among the top 10 images, and one percent higher using binary images. AmphIdent ranked 96.9% and 98.3% of all known matches as the top and among the top 10 images, respectively. Wild‐ID outperformed I3S in three of the four databases, and I3S was better than Wild‐ID in the image database of the fire salamanders. APHIS using binary images generally performed better than when using color images. APHIS outperformed I3S and Wild‐ID in the fire salamander database and performed similar to I3S with the other databases.

**Table 3:** Obtained rank CDF values for the algorithms in the investigated databases. The numbers in the cells are *cdf*(1) and *cdf*(10), that is, the ratio of images that are ranked at top and among the top ten images, respectively

Salamandra spec.	DBSize	500	2,000	2,197		
	AmphIdent	.984/.998	.984/.998	.983/.998		
	I3S	.536/.761	.520/.737	.519/.734		
	Wild‐ID	.137/.268	.118/.230	.116/.226		
	APHIS Color	.750/.854	.745/.844	.744/.843		
	APHIS BW	.832/.884	.830/.881	.830/.880		
Triturus spec.	DBSize	500	2,000	4,000	7,000	7,458
	AmphIdent	.999/1.0	.999/1.0	.999/1.0	.999/1.0	.999/1.0
	I3S	.671/.845	.667/.838	.663/.827	.659/.815	.658/.813
	Wild‐ID	.726/.854	.700/.839	.676/.831	.648/.814	.645/.812
	APHIS Color	.751/.833	.734/.817	.723/.801	.714/.794	.713/.794
	APHIS BW	.800/.871	.792/.863	.788/.857	.784/.846	.784/.845
Ambystoma opachum	DBSize	500	2,000	4,000	7,000	12,488
	AmphIdent	.960/.989	.944/.989	.929/.986	.912/.983	.896/.973
	I3S	.296/.484	.218/.423	.182/.385	.151/.343	.126/.308
	Wild‐ID	.650/.823	.551/.783	.495/.736	.447/.696	.396/.659
	APHIS Color	.450/.595	.409/.527	.388/.493	.372/.474	.363/.456
	APHIS BW	.464/.602	.413/.558	.385/.526	.368/.487	.352/.462
Bombina variegata	DBSize	500	2,000	4,000	4,063	
	AmphIdent	.977/.988	.973/.983	.969/.983	.969/.983	
	I3S	.845/.920	.821/.902	.804/.886	.804/.886	
	Wild‐ID	.953/.973	.941/.967	.932/.964	.932/.964	
	APHIS Color	.875/.915	.867/.904	.863/.896	.863/.896	
	APHIS BW	.886/.927	.879/.914	.873/.907	.873/.907	

### 3.2 Recognition rates by image database size

We found that recognition rate decreased with larger database sizes, but performance decreased differently between photo‐matching algorithms (Figure 3 and Table 3). Recognition rates for Wild‐ID and I3S improved with smaller database sizes and improvement was highest with the marbled salamander database, but did not significantly improve with the toad and fire salamander databases. Recognition rates of I3S only decreased slightly with the newt database size, while Wild‐ID significantly decreased with a larger newt database size. Interestingly, with the newt database, I3S performed better than Wild‐ID with the complete database, but the opposite was true with the smallest database size (500 images). AmphIdent was least sensitive to changes in database size as it performed well with the complete databases; however, a slight performance decrease was seen with growing database size (Figure 3 and Table 3). Compared to I3S and Wild‐ID, APHIS was less sensitive to increasing database size.

### 3.3 Recognition rate by image ranking

We found recognition rates to improve, sometimes dramatically, when evaluating the 10th or higher ranked image compared with just the top ranking image (Figure 3 and Table 3). Performance increases were greatest with the marbled salamander database. With the marbled salamander database, I3S recognition rate increased from 12.6% when only the top ranked image was considered, to 30.8% when considering the top 10 ranked images. In the same database, the performance of Wild‐ID could be improved from 39.6% to 65.9% when considering the top 10 instead of only the top‐ranked image. AmphIdent performance was improved from 89.6% to 97.3% (Figure 3 and Table 3). For APHIS, recognition rate improved from roughly 36% to 46%, regardless of whether colored or binary images were used. In general, the biggest improvement was found with the algorithms that performed poorly when only considering the top ranked image. However, considering the curves in Figure 3, the curves for APHIS are not as steep as the curves of I3S and Wild‐ID. Hence, sometimes the curves cross, showing that APHIS can perform better than other algorithms when considering the top rank only, but perform poorer when considering the top 10 ranked images.

### 3.4 Recognition rate by number of matching images

The overall performance improved greatly with an increasing number of available matches in the database. For the yellow‐bellied toad image database, considering the top‐ranked image only, Wild‐ID increased from a 93.2% recognition rate with one matching image to 100% recognition rate when three matching images are available in the database (Figure 3). However, due to the poor recognition rate in the fire salamander image database, even with three matching counterparts in the database, Wild‐ID achieved only a 30% recognition rate with the top‐ranked match and 49% when considering the top 10 ranked images. I3S also improved based on the number of matches in the image database, as it achieved 92% and 97% recognition rate when evaluating the top ranked image with three existing matches in the database of fire salamanders and toads, respectively. The performance of APHIS using binary images improved to 97% and 98% in the salamander and toad database, and to 95% and 97.5% when using colored images when considering the top rank only, respectively. AmphIdent performance only slightly increased with more matching images in the database as recognition rate was already nearly 100% with a single matching image (Figure 3).

## 4 DISCUSSION

Our results show that performance can differ, at times substantially, depending on photo‐matching algorithm used, database, database characteristics (e.g., image quality and numbers of matching images in the database), and the number of ranked photos evaluated. To our surprise, many of the photo‐matching algorithms had recognition rates with our amphibian databases that would not be acceptable for use in subsequent demographic analyses. Our results also highlight the need to first manually measuring recognition rates (potentially with multiple photo‐matching algorithms) of known visually matched images prior to selecting a specific photo‐matching algorithm and automating the photo‐matching process.

### 4.1 Photo‐matching algorithm performance

Photo‐matching algorithms can be categorized by their use of pixel‐based (AmphIdent and APHIS) or feature‐based algorithms (I3S and Wild‐ID). Our results show a remarkable performance difference between the individual algorithms; however, a clear superiority of one algorithm group was not observed.

**Figure 4:**
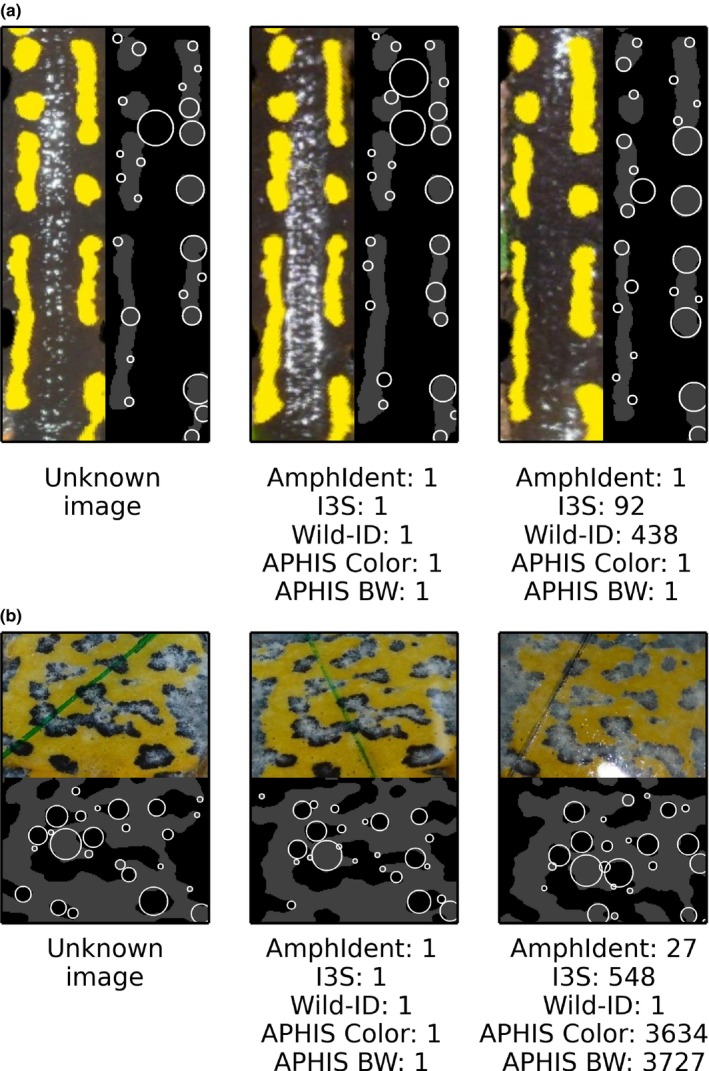
A representative image of an individual fire salamander (a) or yellow‐bellied toad (b) with two matching images. Within each subplot, the colored image is the original image, whereas the gray image is the binarized version overlaid with the key points that were detected by I3S(circles). The numbers indicate the retrieved rank of the matching images with the different photo‐matching algorithms

The issue with photo‐matching algorithms that are based on local features is that they require the local patterns to be very stable among matching images. Variability in patterns between matching images is typically not due to an individual's pattern changing through time [Ferner (2010); Mettouris, Megremis, and Giokas (2016), but see Drechsler et al. (2015) and Kenyon, Phillott, and Alford (2010)] but a result of slight differences due to animal posture, hormone status, injury, environmental influences, or even dirt (Jorgensen & Larsen, 1960; Kindermann, Narayan, & Hero, 2014). Additionally, glare, focus, camera angle, and flash can also cause small perturbations between the images. For example, the three matching images of a fire salamander shown in Figure 4a are ranked at 1st, 92nd and 1st, 438th by I3S and Wild‐ID, respectively, even though the patterns visually appear very similar. Slight variations among the spots occur, especially on the rightmost pattern. These subtle differences in the pattern influence the feature detectors to consider different key point locations. The extracted key points from I3S show that the leftmost and center image share a great amount of similarly located points, while the key points in the rightmost pattern differ, and therefore, this matching pattern is not ranked highly by I3S and Wild‐ID, despite its very similar appearance. Note that the pixel‐based algorithms AmphIdent and APHIS scored both images as a match.

Wild‐ID outperformed I3S in all databases except the fire salamander image database, even though the SURF feature detector of I3S is thought to be more robust than SIFT, which is used by Wild‐ID (Bay et al., 2006). The poor performance by I3S can mainly be explained by a shortcoming of the I3S matching algorithm: I3S solely uses the locations of identified local features to find an affine transform that matches the feature positions of one image to the positions on other images. However, it ignores a numeric characterization of each detected feature point provided by SURF, and hence, it considers two patterns to match, even when their feature descriptors have different values. Wild‐ID instead performs a search for matching feature descriptors first and subsequently evaluates the distances between the locations of these features. This additional processing improves performance of Wild‐ID compared to I3S and explains the superiority in recognition rates of Wild‐ID over I3S in our study. The poor performance of Wild‐ID in the fire salamander database can be explained by the significant amount of glare in the fire salamander images. Wild‐ID, which works directly on the color images, considers these glare regions as important features. However, as glare is not stable between matching images, Wild‐ID performance is degraded. In contrast, I3S works on the binary patterns where the glare was removed by the binarization operation.

A pixel‐based algorithm does not rely on specific key points in the images. Instead, it considers the images as a whole and is hence more robust to subtle changes. Both APHIS and AmphIdent calculate a cross correlation between the pixels of the compared patterns, which can equalize for deformation of spots, as long as the overall appearance of the images are similar. Pixel‐based algorithms rely on a consistent cropping of the pattern, such that matching spots of two patterns occur at (roughly) the same position in the images. In order to equalize for different spot positions that can occur due to animal posture or the cropping region, both AmphIdent and APHIS divide the entire patterns into small parts and compute the cross correlation for each part separately. By employing the cross‐correlation calculation, the overall matching success is not degraded when two matching spots have slightly different appearances: As long as they share a decent amount of similar pixels, the cross‐correlation will yield high similarity scores, indicating that both images belong to the same individual. In addition to individual translations of each part, AmphIdent allows individual scaling of each part, which explains the superior result of AmphIdent compared to APHIS.

Figure 4b shows example images of a yellow‐bellied toad, where the pixel‐based algorithms were not able to reliably retrieve the matching image. Considering the left‐most image as the unknown image, all algorithms retrieved the center image at the top rank. Comparing the left and right images, it becomes apparent that the cropped regions of interest are very different. Therefore, the pixel‐based algorithms do not reliably recognize this match. On the other hand, the feature‐based algorithm Wild‐ID positioned the matching image at rank 1, as it could identify the characteristic shape of the pattern in the image.

### 4.2 Number of matching images in the database

Recognition rates greatly improved for all photo‐matching algorithms when images had more than one matching image in the database. This is because a correct match is counted whenever at least one matching image occurs in the top‐ranked images. Hence, when multiple matching images are available in the database, each algorithm has several chances to measure a high similarity score for a matching image, making it more likely that one matching image occurs among the top‐ranked images. Note that normally, having two matching images in the database implies that a previous match was already found with only a single matching image in the database. Hence, the performance with a single‐matching image limits the overall performance and is therefore a more meaningful and direct measure of algorithm performance. Alternatively, as reported by Sannolo et al. (2016), two images of the same individual could be taken and directly integrated into the image database, because the match is known *a priori*. However, this approach requires increased effort with image preprocessing and image database management, rendering it impracticable for large‐scale databases.

### 4.3 Comparison with previous studies

The recognition rates that we observed for Wild‐ID and I3S appear to contradict to several published results. Mettouris et al. (2016) reported a 100% recognition rate for alpine newts (*Ichthyosaura alpestris*) and smooth newts (*Lissotriton vulgaris*) when using Wild‐ID with a database of 3,333 images. However, images were sorted into four classes depending on gender and species, yielding very small databases (162, 136, 26, and 13 images per class) for the photo‐matching evaluation, and the overall number of matching image pairs was only 25.

Bendik, Morrison, Gluesenkamp, Sanders, and O'Donnell (2013) used Wild‐ID to match images of Jollyville Plateau salamanders (*Eurycea tonkawae*) and reported a recognition rate of 99.3% with a database of 1,367 images. However, recognition rate was based on a rank of 100 or better which is beyond the ranking considered in our study and is not a realistic number of images to review, with a large database.

Wild‐ID has also been previously used to match images of the Wyoming Toad (*Anaxyrus baxteri*) with the authors reporting a recognition rate of approximately 53%, even with a small database size (Morrison et al., 2016). Hence, the performance of Wild‐ID based on our research and previous research performance of Wild‐ID can significantly differ between databases and a thorough evaluation of recognition rate is important prior to matching a complete image database with Wild‐ID.

I3S Classic has previously been used to match images of the common wall lizard (*Podarcis muralis*) and western green lizard (*Lacerta bilineata*) with a recognition rate of 99% with a database of 1,043 images (Sacchi et al., 2010). In contrast to I3S Pattern+, I3S Classic requires the user to manually set the key points instead of employing an automatic feature detector as in I3S Pattern+. This requires a significant amount of manual processing, but by defining a consistent rule for setting the points, a better identification rate can be obtained.

I3S Pattern was used to match images of Italian crested newts and found a 100% recognition rate with a database of 852 images (Sannolo et al., 2016). In this study, the database was structured in a way that at least three matching images for each unknown image were included in the database. With this amount of redundancy in the database, a recognition rate of 100% is feasible considering that recognition rate greatly improves with multiple matching images in a database.

APHIS was used in (Oscar et al., 2015) to match 309 images of the Northern spectacled salamander (*Salamandrina perspicillata*) including 19 recaptures and 287 images of the Balearic lizard (*Podiarcis lilfordi*) including 91 recaptures, where it achieved a matching rate of 100% and 93.4%, respectively. A correct match was counted, when the match occurred among the top 20 ranked images. These results are roughly in line with the results obtained in this study for the toad database, considering the small database sizes.

In (Drechsler et al., 2015), the authors used AmphIdent to match a database of 1,648 images of high quality with 162 recaptures of the great crested newt and obtained a recognition rate of 98% when manually comparing the 10 highest ranked images. Moreover, the authors in (Goedbloed et al., 2017) used AmphIdent to compare images of *Salamandra infraimmaculata*, obtaining recognition rates of 100% for high‐quality images and 64.8% for very poor quality images. The results for high‐quality images are in line with this work.

### 4.4 Implications of high false rejection rates for population models

Unfortunately, even relatively low FRRs have been found to bias estimates of vital rates and population size (Morrison et al., 2011, 2016; Winiarski & McGarigal, 2016). For example, Morrison et al. (2016) reported a 200% positively biased population size with a FRR of 21.3%. Statistical approaches have been developed to incorporate false rejection error (FRE) with CR data (Givens et al., 2015; Hiby et al., 2013; Morrison et al., 2011). Although potentially flexible, many existing statistical approaches incorporating FRE focus on estimating population size, rather than survival, with closed population models and are not easily incorporated with existing CR software packages. To calculate accurate estimates of survival, it is recommended to have FRRs no greater than 5% as slight bias in survival estimates, especially adult survival of long‐lived species, can significantly bias estimates of population growth (Winiarski & McGarigal, 2016). A more rigorous analysis of the implications of misidentification on the population models is out of scope of this work; we refer the reader to dedicated publications, such as Pradel, Hines, Lebreton, and Nichols (1997); Creel et al. (2003); Yoshizaki, Pollock, Brownie, and Webster (2009); Wright et al. (2009); Link, Yoshizaki, Bailey, and Pollock (2010).

FRR for a given image database can be estimated by collecting matches from visually matching a subset of images and then using a selected photo‐matching algorithm to measure similarity scores between all images in the database. This allows FRR to be calculated and gives the user guidance with how many ranked images should be reviewed to obtain the required recognition rate.

## 5 CONCLUSIONS

This study presented a thorough analysis of matching performance of pixel‐based and feature‐based photo‐matching algorithms for amphibian image databases. Even though this presentation was limited to amphibian databases, we believe the obtained results are generalizable to other taxa. None of the investigated algorithms is specifically designed to match amphibians, but their principle can be applied to any spot pattern. We found that the pixel‐based algorithm of AmphIdent outperformed the other algorithms, whose performance varied significantly by image database. Further, algorithm performance depended on image characteristics, number of reviewed images, and the number of available matches in the database. Hence, researchers should show care in selecting a photo‐matching algorithm which maximizes recognition rate. Improving recognition rate will improve demographic estimates and enables the use of very large databases which are unfeasible or virtually impossible to visually match.

## CONFLICT OF INTEREST

Maximilian Matthé is the developer of the commercially available software AmphIdent. The other authors declare no conflict of interest.

## AUTHOR CONTRIBUTIONS

MM conceived the idea, analyzed the data, and led the writing of the manuscript. MS, KW, AS, DG, SS, US collected the data and processed the obtained images. All authors contributed critically to the drafts and gave final approval for publication.

## DATA ACCESSIBILITY

The obtained similarity scores for all databases will be published, along with the source code that was used to perform the analysis. All image databases from this work are publicly available. Additionally, the modifications of I3S and Wild‐ID to make them able to perform batch processing are freely available. A web interface for double checking the results from AmphIdent is available at http://www.amphident.de/aiOnline.html.
